# Methicillin-Resistant *Staphylococcus aureus* ST398, Italy

**DOI:** 10.3201/eid1602.091478

**Published:** 2010-02

**Authors:** Laura Soavi, Roberto Stellini, Liana Signorini, Benvenuto Antonini, Palmino Pedroni, Livio Zanetti, Bruno Milanesi, Annalisa Pantosti, Alberto Matteelli, Angelo Pan, Giampiero Carosi

**Affiliations:** University of Brescia, Brescia, Italy (L. Soavi, R. Stellini, L. Signorini, A. Matteelli, G. Carosi); Presidio Ospedaliero di Manerbio, Manerbio, Italy (B. Antonini, P. Pedroni, L. Zanetti, B. Milanesi); Istituto Superiore di Sanità, Rome, Italy (A. Pantosti); Istituti Ospitalieri di Cremona, Cremona, Italy (A. Pan)

**Keywords:** MRSA, infection, cattle, Staphylococcus aureus, bacteria, Italy, letter

**To the Editor:** It has recently become apparent that livestock can constitute a new methicillin-resistant *Staphylococcus aureus* (MRSA) reservoir and be a source of a novel and rapidly emerging type of MRSA. These livestock-associated MRSA clones are nontypeable by use of pulsed-field gel electrophoresis with *Sma*I and belong to sequence type (ST) 398 (*1*). MRSA ST398 clones account for 20% of all MRSA in the Netherlands (*2*), but the emergence of such clones has been described worldwide (*3*). Although ST398 transmission has been reported primarily between animals, persons with occupational exposure to livestock are at higher risk for MRSA carriage than the general population. Even though MRSA ST398 usually causes colonization, several cases of infections of variable clinical relevance, varying from skin and soft tissue infections (*4*) to endocarditis (*5*) and pneumonia (*6*), have been described over the past few years. Most instances of ST398 human carriers have been identified among persons who work at pig farms (*7*). Data regarding MRSA colonization of dairy farmers are less exhaustive and, to our knowledge, only 1 instance of direct transmission between cattle and humans has been proven. MRSA isolates from cows with subclinical mastitis in 2007 in Hungary were indistinguishable from MRSA isolates from the tonsil swab of a farmer who worked with these animals (*8*). We report a case of MRSA ST398 invasive disease in a cattle farmer, as well as a case of MRSA ST398 necrotizing fasciitis.

In early April 2008, a 52-year-old man was admitted to an intensive care unit in Manerbio, Italy, because of severe sepsis and a large ulcerative and suppurative lesion on the right side of his neck. His medical history was unremarkable. He was a worker at a dairy farm, was obese, and did not report any previous contact with the healthcare system. At the time of hospital admission, he was oriented and cooperative. His temperature was 38.4°C, heart rate was 125 beats per minute, and blood pressure was 165/75 mm Hg. Arterial blood gas analysis showed hypoxemia and mild hypocapnia (PaO_2_ 53 mm Hg and PaCO_2_ 33.8 mm Hg on room air). Leukocyte count was 21,280 cells/μL (81.9% polymorphonuclear cells), and platelet count was 310,000 cells/μL. After blood samples were collected and aggressive surgical debridement of affected tissue was performed, empirical treatment with intravenous teicoplanin and imipenem was started. On the basis of histologic appearance of the intraoperative material and computed tomography scan images, necrotizing fasciitis was diagnosed. Culture of blood and necrotic tissue yielded MRSA. On day 3 after admission, antimicrobial drug therapy was changed to teicoplanin and clindamycin and, on day 7, to linezolid. Fever resolved in 3 days and the patient’s condition progressively improved. The patient was discharged after 31 days of antimicrobial drug therapy. The MRSA isolate was susceptible to all the non–β-lactam antimicrobial drugs tested (excluding tetracycline), carried the staphylococcal cassette chromosome *mec* type V, and was negative for Panton-Valentine leukocidin (PVL) genes. Multilocus sequence typing and sequence typing of the tandem repeat region of protein A gene (*spa* typing) showed that the isolate belonged to ST398 and *spa* type 899, respectively.

Some issues are of concern. Although the MRSA isolate was PVL negative, its virulence resembled that of PVL-positive strains. Furthermore, it was resistant to tetracycline, as we expected because oxytetracyclines are the antimicrobial drugs most frequently used in pig and cattle farming (*3*). The major limitation of our study was that data regarding MRSA colonization of the farm are missing, so cattle-to-human transmission cannot be proven. However, because our patient did not have any other potential risk factor, dairy cows were probably the source of the human infection. In countries where community-acquired MRSA is common, all patients with serious *S. aureus* infections should be treated for MRSA until antimicrobial susceptibilities are known. Our report suggests that even in countries where community-acquired MRSA is still rare, being a cattle farmer may be considered an indication for early treatment against MRSA.

The expanding knowledge of this zoonotic potential may undermine existing nosocomial MRSA control programs. In countries where a search and destroy policy (*9*) is adopted, such as the Netherlands, pig and cattle farmers may warrant screening and isolation at the time of hospital admission. Nevertheless, the first MRSA ST398 nosocomial outbreak has already been described (*10*).

It is difficult to prevent persons with constant exposure to MRSA in their work or home setting from becoming MRSA carriers. Revisiting policies for the use of antimicrobial drugs on livestock farms, as well as improving hygiene measures, may therefore be necessary in infection control programs. However, before final recommendations can be made, further investigation is needed to determine the prevalence of MRSA among livestock and their handlers.
